# Evaluation of the participatory rural appraisal training–hands-on for medical (PRATHaM) students

**DOI:** 10.3389/fpubh.2026.1870942

**Published:** 2026-08-21

**Authors:** Saurabh RamBihariLal Shrivastava, Gandes Retno Rahayu, Titi Savitri Prihatiningsih, V. Pragadeesh Raja

**Affiliations:** 1 Department of Community Medicine, Datta Meghe Medical College, Datta Meghe Institute of Higher Education and Research, Nagpur, Maharashtra, India; 2 Dr. D. Y. Patil School of Allied Health Sciences, Dr. D. Y. Patil Vidyapeeth, Pune (Deemed to be University), Pimpri, Pune, Maharashtra, India; 3 Department of Medical Education and Bioethics, Faculty of Medicine, Public Health, and Nursing, Universitas Gadjah Mada, Yogyakarta, Indonesia; 4 Department of Community Medicine, Father Muller Medical College, Kankanady, Mangalore, Karnataka, India

**Keywords:** community-based education, force field analysis, medical students, participatory rural appraisal, social mapping

## Abstract

**Background:**

Training in conventional community medicine often relies on didactic lectures and limited community engagement, which may not adequately improve understanding of students of contextual sociocultural and health-related problems. Participatory rural appraisal (PRA) is an effective and efficient approach to deliver community-based education. The objective of the present study was to evaluate the Participatory Rural Appraisal Training – Hands-on for Medical (PRATHaM) program for the first-year undergraduate medical students by assessing knowledge acquisition and obtaining feedback from students, faculty members, and community stakeholders.

**Methods:**

It was a quasi-experimental study with a one-group pretest-posttest study design conducted among first-year undergraduate medical students in a medical college. The study employed total sampling, in which 250 students were enrolled, and data from 245 completers were analyzed. Study was carried out in two stages, namely training of students in PRA methods in classroom, followed by implementation of the same in the community by the trained students. At each stage, students were asked to provide feedback on different aspects of the training, and feedback was obtained from the community as well. Two tools were used to measure feedback from the community, namely observation of students’ interaction in the community and feedback from community about their interactions with medical students. Institutional Ethics Committee approval was obtained prior to the start of the study and students were assured that the study will not have any impact on academics. The statistical analysis was done using descriptive and inferential (paired *t*-test) statistics and qualitative data from open-ended questions were analyzed using the thematic analysis framework.

**Results:**

A total of 245 students with a mean age of 20 ± 2.3 years were trained in PRA methods (remaining five were absent). Out of a maximum score of 10, there was a significant improvement in the mean score of students in the post-test (8.80 ± 1.01), when compared with the mean scores in the pre-test (3.47 ± 1.06), and this difference was found to be highly statistically significant (*t* = 56.98, *p* < 0.001). The community members liked the friendly approach adopted by students and that they shared their personal numbers to get in contact in case of any medical issues. The employment of interactive teaching methods, opportunities to interact with local community and implement PRA methods and practical sessions were identified as the most encouraging factors. However, not being aware of the native language, and limited time to interact with allotted families were recognized as the major barriers.

**Conclusion:**

In conclusion, the training imparted to medical students on selected PRA methods brought about a statistically significant improvement in the knowledge of medical students. The findings suggest that PRA-based training is an effective approach for improving medical students’ knowledge and community engagement skills and supports its integration into undergraduate community medicine curriculum to strengthen community-based learning experiences.

## Background

The transformation of medical education in the 21st century demands an institutional shift toward Community-Based Education (CBE) to foster students’ sociocultural competencies. CBE is an approach in which members of the community participate to deal with the local concerns, and in the process, there is practical application of knowledge (1). In the global context, the delivery of CBE plays an instrumental role in augmenting the socio-behavioral attributes of undergraduate medical students (1). In-fact, CBE aids the student to enhance their perception and understanding pertaining to the sociocultural attributes relevant to individual level, family level, and community level at large (2). The implementation of CBE in rural settings has become quite vital due to persistent rural–urban health disparities and shortage of healthcare professionals (2). If we can expose medical students to sociocultural determinants of health at an early stage, we can positively motivate them to serve in underserved rural areas willingly (2).

Moreover, considering the fact that the ultimate purpose of medical education is to benefit the general population, we have to take specific measures to deliver context-specific education (3–5). Further, the findings of multiple studies have shown better attainment of intended learning outcomes in heterogeneous settings (6, 7). Participatory rural appraisal (PRA) is a specific type of community-based approach for carrying out research and planning, which essentially includes active involvement of the local community in identifying their own concerns and making appropriate decisions (8, 9). A number of such tools like transect walk, social mapping, force field analysis, etc., have been used worldwide and have shown immense potential to deliver encouraging results (10–16). These methods actively engage students with the community, facilitate observation, critical thinking, problem-solving, and collaborative decision-making, and simultaneously also inculcate empathy and a deep understanding of the social health determinants (10–16).

From the perspective of delivery of community-based (contextual) curriculum to first-year undergraduate medical students, these PRA methods have massive scope and utility to establish a liaison with the local community by making them aware of the demographic attributes, sociocultural practices, and the prevailing problems in the region (8, 9, 13). As all these tools are visual and easy to employ, students remain actively engaged in the complete process and learn by doing (8, 10, 15, 17). The employment of PRA methods in the first year of training not only helps medical educators to meet the standards of early clinical exposure by the regulating body but even makes the learning joyful and meaningful (8, 9).

The exposure of medical students to participatory rural appraisal methods within the curriculum benefits all the involved stakeholders, namely teachers, students, administrators, community (8, 9, 17, 18). Further, this kind of exposure in the community can be the stepping stone to motivate medical students of the current generation to serve the rural community, and resolve the problem of shortage of healthcare professionals in rural settings (19–21). Although the utility of PRA has been widely demonstrated in the macro-development sector, the literature on its structured application within the core medical education curriculum remains very limited. (9, 22). Current research generally focuses on the public health outcomes resulting from PRA interventions, but neglects pedagogical evaluation of how medical students assimilate these PRA skills.

Although PRA methods have been widely employed in community development and public health programs, limited evidence is available about its structured integration into undergraduate medical curriculum, especially in the early training phase. Similarly, shortage of studies has been reported, wherein evaluation of a comprehensive training model that combines classroom instruction and supervised field implementation. At the same time, minimal studies have examined the perspectives of multiple stakeholders to assess both educational effectiveness and the acceptability of such training programs. To address all these gaps, the present study developed and evaluated the Participatory Rural Appraisal Training–Hands-on for Medical (PRATHaM) program, designed for first-year undergraduate medical students.

The conceptual framework for the PRATHaM program integrates principles of CBE (provides a learning environment in which students engage directly with communities to understand health determinants in real-world settings), PRA, experiential learning theory, and the Kirkpatrick Model of training evaluation. Within this framework, PRA serves as the experiential learning strategy in alignment with the Kolbs’s experiential learning theory by actively involving students and community members. The effectiveness of this educational approach was evaluated using level 1 (participants reaction), level 2 (change in knowledge), and level 3 (change in behavior) of Kirkpatrick Model. This study addresses these gaps by evaluating the PRATHaM program for the first-year undergraduate medical students by assessing knowledge acquisition and obtaining feedback from students, faculty members, and community stakeholders.

## Methods

### Study design

**Figure 1:**
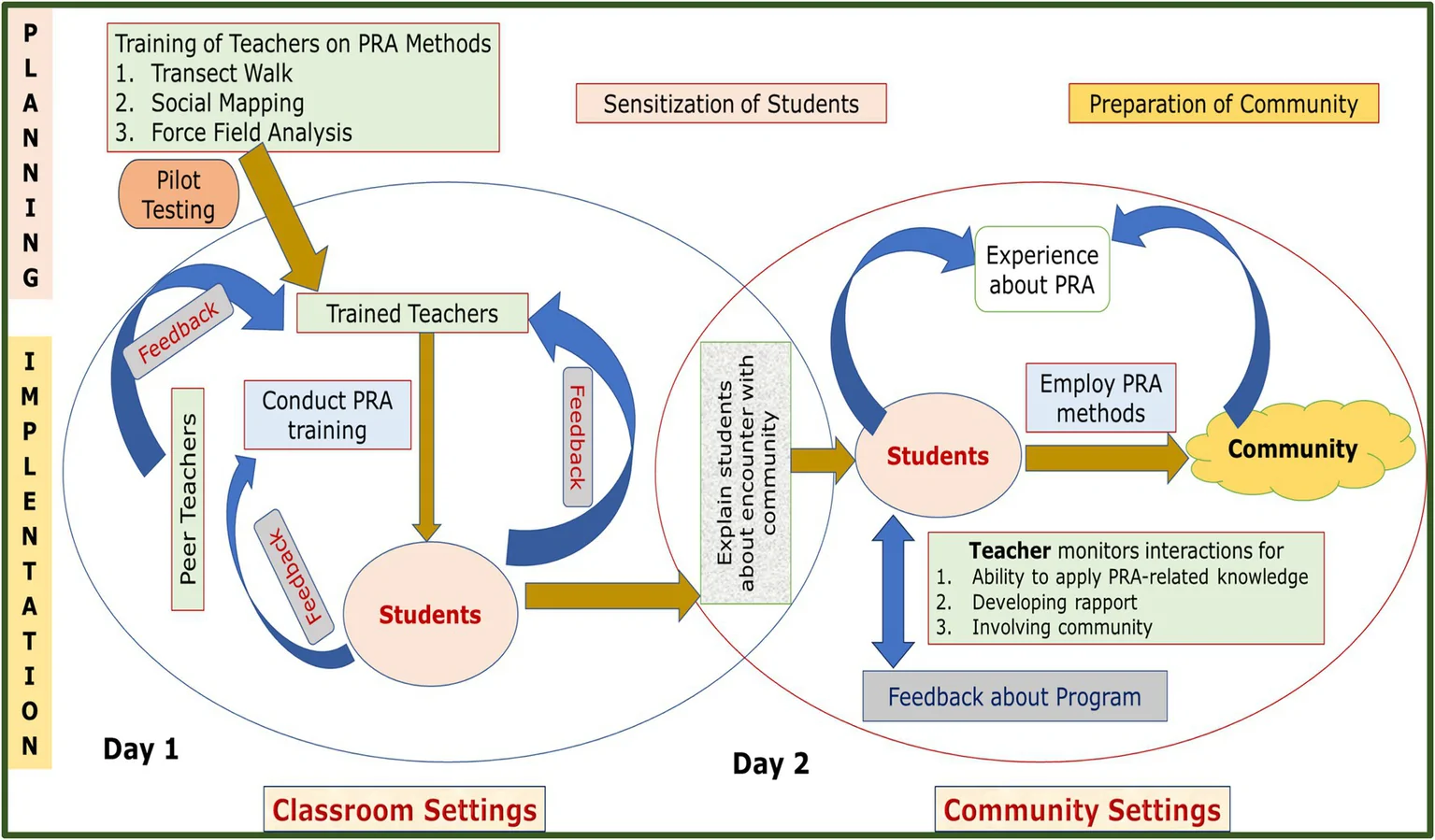
Conceptual framework.

This study employed a quasi-experimental study with a one-group pretest-posttest study design, and was conducted in two stages, namely training of first year undergraduate medical students on PRA methods by the trained teachers on Day 1, followed by implementation of PRA methods in the community by the trained first-year undergraduate medical students on Day 2 (Figure 1). This design was considered appropriate as this program was implemented as a routine educational intervention for the entire cohort of first-year undergraduate medical students. The inclusion of a control group was not educationally appropriate, as withholding structured learning experiences from a subset of students could have resulted in unequal learning opportunities.

### Study setting

In a tertiary medical college and its field practice area.

### Study population

Faculty from the Department of Community Medicine and first-year undergraduate medical students.

### Study participants

A total of five teachers with Master’s Qualification in the specialty of Community Medicine, with teaching experience of 4–10 years in total were involved in the different stages of the training program, in different capacities, namely resource person, peer-evaluator, facilitator, and as an observer in the community. A total of 245 students from the first-year of undergraduate teaching were included in the study, and this training program was held in the month of December 2023, after they all joined institution in October 2023.

For the assessment of change in knowledge, all 250 students were included in the study, while for the assessment in change in behavior, 250 students were divided into 10 groups of 25 students each. Three groups (75 students) were selected randomly using simple random sampling by lottery method, and these three groups were observed by the teachers in the community in 3 lanes owing to limited number of assessors.

### Sampling method

Total sampling method for the knowledge component, and simple random sampling for the behavior component was employed.

### Inclusion and exclusion criteria

All first-year students were included as a part of the study. However, those students who remained absent (*n* = 5) during the conduct of the study were excluded.

### Study variables

The study comprises of the following study variables, namely knowledge about PRA methods among students, ability of students to engage (viz. provide clear instructions to the community, establish rapport, motivate community to participate in PRA methods, etc.) community.

### Data collection tools

A few tools were used in the present study to comprehensively assess and evaluate different aspects of the training as well as evaluation of the complete programme. The questionnaire was developed following a literature review and aligned with the learning objectives of the PRATHaM programme. The draft items were reviewed by two internal and two external subject experts for relevance, clarity, and comprehensiveness, revised based on their feedback, and finalized prior to implementation. These tools include a semi-structured questionnaire to obtain feedback from students about the training they received from their teachers (Kirkpatrick level 1 evaluation), Pre-Test and Post-Test (Kirkpatrick level 2 evaluation with the help of 10 multiple choice questions (MCQs) with one best possible answer), feedback from peer teachers, observation of students’ interaction in the community (Kirkpatrick level 3), feedback from students about their community interactions, feedback from community about their interactions with medical students. Some of the representative items, include which PRA tool is used for resource mapping? What is the purpose of a transect walk? State one principle of PRA, etc. For pre-test and post-test tool, the item-content validity index (0.86), scale-content validity index (0.84), and Cronbach’s *α* (0.88).

### Methodology

The training program schedule was developed after taking input from an external subject expert who was also involved in the act of training department teachers. Training program was conducted in two stages, spread across 2 days, in which the first day was earmarked for training of students, while on the second day, students implemented PRA methods in the community (Figure 1). We must note that prior to the start of the actual study, in the planning stage, we conducted training of teachers and postgraduate students in the department, of which teachers were subsequently involved as resource person for training the first-year undergraduate medical students in the first stage. The training of teachers was done with the help of an external expert and primary investigator. To enhance the effectiveness of training, we conducted a pilot-run of the entire training session in the planning phase, wherein the target students were from the second-year medical students (Figure 1). This pilot run was followed up by receiving feedback from the students, which in turn gave us valuable insights about the common mistakes. Accordingly, the training workshop was revised to enhance the effectiveness of the training sessions.

Also, to reduce the bias in the studies, the primary investigator was only involved in the training of teachers. All the teachers and postgraduate residents were trained in all data collection instruments to avoid any kind of inter-observer variations. First-year undergraduate medical students were sensitized about the entire training program and then trained in different PRA methods using a combination of teaching-learning methods. These training sessions were facilitated by trained teachers, and each of the sessions was peer-evaluated as well. For the current study purpose, a total of three sessions of 45 min duration each were taken by three different teachers. After the completion of the three sessions, a Google feedback form was circulated to obtain views of participating students on the assessment of teachers, the content of the session, and the supportive aspects (Kirkpatrick Level 1 evaluation). All students were trained in a single training session in a large group to ensure uniformity. Standardized checklist or data collection instrument was used by all 3 observers, while inter-rater reliability was not measured.

### Field implementation and group allocation

On the first day of the training program, all the undergraduate students were divided in 10 groups, comprising 25 each, except the last group that had 20 students. Each of these groups had 6–8 students who could speak Tamil language, and to support non-Tamil speaking students, a multi-lingual vocabulary was shared with them. Each of these groups were allocated one faculty member, one postgraduate student, and four interns, and all these small groups had one-to-one meetings. The teachers explained the purpose of the group and assigned responsibilities to postgraduate students and the interns, wherein they had to facilitate the learning process of students. Students were explained regarding their task, and they were reassured of constant support during the community visit, wherein they must implement PRA methods that were told to them, including the steps that need to be followed to attain better engagement of students. Further, for each of the groups, specific lanes in Kothimangalam village were allocated, and students were informed to implement PRA methods among those families that are living in the allocated lanes only to avoid overlaps.

### Student observation in community

On Day 2 of the program, students were taken to the allotted lanes in the Kothimangalam village and then briefed once again about the task that need to be carried out. The field activities started with transect walk, wherein all the group members had a purpose-driven walk across the allocated lanes to understand the important infrastructure and facilities. Subsequently, students approached the local villagers, preferably women (as most men have gone for their work) to draw social mapping on the ground using locally available items. In addition, some of the members from each student group divided themselves into groups of 3–4 to perform force field analysis on the theme-the promoting and restraining factors in accessing healthcare services. Of the 10 groups, three groups were randomly selected using a lottery method (simple random sampling), and their interactions with the members of the community were constantly observed by teacher. It is important to state that even the students of the remaining seven groups were observed during their interactions, but they were not included as a part of the current study due to teacher shortage, operational and logistic constraints.

### Statistical analysis

The data collected across different stages of the study was analyzed using descriptive statistics (mean, standard deviation, frequency, and percentages) and inferential statistical methods. The collected data was analyzed using SPSS version 16.0. The statistical differences between pre-test and post-test scores were evaluated using a paired t-test with a significance level set at *p* < 0.05. In addition, the mean difference with 95% confidence interval and Cohen’s *d* effect size were calculated to assess the magnitude and practical significance of the intervention. Qualitative data from open-ended questions were analyzed using the thematic analysis framework by Braun and Clarke to identify recurring codes and themes. At the beginning, all responses were reviewed independently by two investigators, and similar responses were inductively coded and grouped into common themes through discussion and consensus. As there was a possibility that individual participants could mention multiple ideas in a single response, each response was assigned to one or more relevant themes. The frequency and percentage of participants mentioning each theme were then calculated; therefore, the responses were not mutually exclusive.

### Ethical considerations

Permission from the Institutional Human Ethics Committee was obtained prior to the start of the study. The assigned registration number was 2023/806, dated 26 July 2023. Written informed consent was obtained from the students as well as the members of the community before they were included in the study. In addition, it was communicated to the students that the responses obtained from the students will be kept confidential and will have no impact on their academics.

## Results

The mean age of participating students was 20 ± 2.3 years, with most of the students being female [149 (60.8%)], and rest were male [96 (39.2%)]. Out of 245 students, 80 were from Tamil Nadu (knowing the native language–Tamil, which the residents speak), while 165 students had limited or no command over Tamil language, and thus they were consciously grouped with Tamil-speaking students for community interactions. Community participants comprised adults and older people who were available during the scheduled community visits, and they participated voluntarily to facilitate the implementation of PRA activities. As the primary focus of the present study was to evaluate the educational intervention, detailed information of community participants was not systematically collected.

### Training students on participatory rural appraisal methods (knowledge component)

The findings of the feedback form have been depicted in Table 1. In the same feedback form, we asked two open questions related to training attributes that were liked by the students (Table 2), and areas that need improvement (Table 3).

**Table 1:** Evaluation of classroom training.

Teacher assessment	Excellent	Very good	Average	Weak	Extremely Weak
Academic proficiency of the trainer	185 (75.5%)	51 (20.8%)	9 (3.7%)	—	—
Ability to transfer the concepts to learners	138 (56.3%)	92 (37.6%)	15 (6.1%)	—	—
Class management abilities	125 (51%)	107 (43.7%)	13 (5.3%)	—	—
Use of interactive teaching methods	152 (62%)	70 (28.6%)	23 (9.4%)	—	—
Ability to respond to the queries	221 (90.2%)	21 (8.6%)	3 (1.2%)	—	—
Frequency of using Practical examples during teaching	179 (73%)	61 (25%)	5 (2%)	—	—

Content Assessment	Strongly agree	Agree	Neutral	Disagree	Strongly disagree
Mentioning specific learning objectives (SLOs) at the start of the session	243 (99.2%)	2 (0.8%)	—	—	—
Alignment between SLOs and delivered content	129 (52.7%)	104 (42.4%)	12 (4.9%)	—	—
Provision of updated content	137 (55.9%)	92 (37.6%)	16 (6.5%)	—	—
Content effectiveness in increasing your knowledge	201 (82%)	37 (15.1%)	7 (2.9%)	—	—
Citing examples from the field	183 (74.7%)	57 (23.3%)	5 (2%)	—	—

Support assessment	Very appropriate	Appropriate	Neutral	Inappropriate	Very Inappropriate
Overall duration of training	109 (44.5%)	99 (40.4%)	35 (14.3%)	2 (0.8%)	—
Optimal mix of theory and practical sessions	103 (42%)	106 (43.3%)	27 (11%)	9 (3.7%)	—
Seating arrangement	142 (58%)	44 (18%)	47 (19.1%)	12 (4.9%)	—
Audio-visual support	118 (48.2%)	67 (27.3%)	23 (9.4%)	26 (10.6%)	11 (4.5%)
Venue arrangement	134 (54.7%)	78 (31.8%)	31 (12.7%)	2 (0.8%)	—

**Table 2:** Training attributes that were liked by students.

Training attributes	Number (%)^*^
Active involvement of teachers and efforts to make students learn	32 (13.1%)
Questioning students during the session, including problem/scenario-based	89 (36.3%)
**Interactive and creative teaching methods** (including WhatsApp polling, YouTube, Visuals / Videos of previous batches, Experience sharing from senior students, Visit to lab for models/charts, etc.)	**178 (72.7%)**
Assignments/activities given during the sessions	145 (59.2%)
Delivery of content in simple words	159 (64.9%)
Tips to interact with community members and explanation of each term	134 (54.7%)
**Comprehensive and engaging content with logical explanation**	**166 (67.8%)**
Clearly specifying what we have to do in community	139 (56.7%)
Friendly teachers	87 (35.5%)
Learning through observation and sharing of experiences	149 (60.8%)
Summary at the end of the session	121 (49.4%)
**Citing real-life examples that made the concept easier to grasp**	**164 (66.9%)**
**Fun-filled learning and interaction with students**	**166 (67.8%)**
Getting to know about the responsibility towards the allotted family	72 (29.4%)
Question-Answer sessions after every topic	55 (22.4%)
Learned new things other than routine theory	97 (39.6%)

*Responses were analyzed using inductive thematic coding. Similar responses were grouped into common themes, and as individual participants could contribute to more than one theme; therefore, responses were not mutually exclusive. Bold values indicate the most common training attributes liked by students.

**Table 3:** Training attributes that need to be improved.

Training attributes	Number (%)^*^
**Audio-visual support (audio not heard sometimes)**	**74 (30.2%)**
Sharing slides for the session	16 (6.5%)
Visibility of content	19 (7.8%)
Some of the sessions were slow-paced	27 (11%)
**More number of fun activities could be given**	**29 (11.8%)**
**More time could have been allocated when discussing activities**	**32 (13.1%)**
Presentation could have more images	22 (9%)
Interaction with students could be better	19 (7.8%)
Better management of students	17 (6.9%)

* Responses were analyzed using inductive thematic coding. Similar responses were grouped into common themes, and as individual participants could contribute to more than one theme; therefore, responses were not mutually exclusive.

Table 3 shows the responses of students about the training attributes that need to be improved in subsequent sessions. 156 (63.7%) students reported expressed absolute satisfaction and they liked all the planned activities.

### Findings from peer observation of teaching

As mentioned earlier, peer observation of teaching was carried out for three didactic sessions that covered the selected three participatory rural appraisal methods (viz. transect walk, social mapping, and force field analysis). These three PRA methods were covered by three teachers in large- group teaching sessions using a combination of teaching-learning methods. The peer observation of teaching for each session was done by another four peer teachers, and the key observations pertaining to the positive aspects in the session, and areas that need improvement in future, have been reported in Table 4.

**Table 4:** Peer observation of teaching.

PRA methods	Strengths and the areas that need improvement	
Transect walk	Positive aspects	Interactive session
		Incorporation of videos
		Experience sharing
	Areas that need improvement	Content can be covered in-depth
		Giving attention to the entire class
Social mapping	Positive aspects	Supporting statements with field examples
		Active engagement of students
		Step-by-step approach to perform social mapping
		Content clarity
	Areas that need improvement	Could have shown the do’s and don’ts
		Could use pre and post-test questions in the session
Force field analysis	Positive aspects	Suitable examples were given
		Interaction encouraged through polling questions
		Content of the slides was good and logical
		Time management
	Areas that need improvement	To add some more pictures
		Mention references at the end of the session

### Pre-test and post-test analysis

Out of a maximum score of 10, there was a significant improvement in the mean score of students in the post-test (8.80 ± 1.01), when compared with the mean scores in the pre-test (3.47 ± 1.06). This difference was found to be highly statistically significant (*t* = 56.98, *p* < 0.001) and was associated with a very large effect size (Cohen’s *d* = 3.64), indicating substantial educational impact (see Table 5).

**Table 5:** Pre- and post-test performance of students.

Parameters	Pre-test	Post-test	Mean difference (95%CI)	*p*-value	Cohen’s d
Mean	3.47	8.80	5.33 (5.15–5.51)	**<0.001**	3.64
Standard deviation	1.06	1.01	1.4642		
Standard error of mean	0.07	0.06	0.094		
Number	245	245			

## Implementation of participatory rural appraisal methods in the community

### Students’ observation in community

Of the 10 groups, three groups were randomly selected using a lottery method (simple random sampling), and while the students from these 3 groups (viz. Group 2, Group 6, and Group 9) were interacting with the members of the community in the village, a teacher was constantly observing the interactions and recorded their findings in the Google Form (Table 6). Table 6 demonstrates the qualitative themes emerging from stakeholder perception regarding implementation of PRA training. The observations from teachers indicated that most students were able to explain the purpose of the interaction, establish rapport, and encourage community participation in the PRA activities. However, there were areas for improvement as well, namely improving communication skills of students, allowing additional time for community interactions, and extending greater support for students experiencing language barriers.

**Table 6:** Qualitative themes emerging from stakeholder perception regarding implementation of PRA training.

Theme	Sub-theme	Stakeholder observations
Student engagement and participation	Active involvement	Students actively participated in PRA activities and demonstrated enthusiasm during community interactions
	Community rapport	Students successfully established rapport with community members and explained the purpose of the activities effectively
Communication and interpersonal skills	Effective communication	Most students communicated respectfully and encouraged participation of community members
	Areas for improvement	Some students required further training to improve communication skills and confidence during community interactions
Implementation of PRA activities	Facilitation of PRA tools	Students were able to conduct PRA exercises with active participation from community members following appropriate guidance
	Operational challenges	Language barriers and limited interaction time occasionally affected implementation of PRA activities
Learning experience	Experiential learning	PRA activities enhanced students’ understanding of community health problems and social determinants of health through direct engagement
Recommendations for improvement	Training needs	Additional orientation in communication skills and PRA facilitation was suggested
	Program logistics	Longer duration of community visits and increased time for interaction were recommended to improve learning outcomes

Some of the quotations from participants include.

#### Theme learning experience (sub-theme: experiential learning)

Students highlighted how the hands-on nature of the PRATHaM program bridged the gap between theoretical knowledge and real-world public health dynamics.

One student noted: *“The village visit helped me understand health problems that cannot be appreciated inside the classroom.”*

#### Theme student engagement and participation (sub-theme: active involvement)

Faculty observers underscored the progressive nature of student engagement and the visible boost in their clinical confidence over the course of the field implementation.

A faculty member observed: *“Students became more confident while interacting with the community as the sessions progressed.”*

#### Theme communication and interpersonal skills (sub-theme: effective communication)

Feedback from the local residents confirmed that the students successfully established a respectful, empathetic, and communicative rapport.

As one community member shared: *“Maanavargal engal kavalaigalaik kavanamaagak kaettu, udalnalam saarntha vishayangazhai yelimaiyaana muraiyil vilakkinaargal” (The students listened carefully to our concerns and explained health issues in a simple manner.”*

### Feedback from community about interaction with students

Upon the completion of interaction between students and community on Day 2, the assigned faculty member from each of the three groups interviewed some of the community members in Tamil language. The purpose of the interview was to just identify factors that aided or inhibited the interaction with the assigned medical students, as depicted in Table 7. The good thing about such interactions was that all the assigned families were already aware that medical students were going to come to meet them and interact with them, as members of the community were sensitized about the interaction prior (Figure 1).

**Table 7:** Perception of different stakeholders on community interactions.

Stakeholders	Promoting factors	Inhibiting Factors
Teachers’ perception	Active participation of students	Need training in communication before community exposure
	Treating community members with respect and humility	More time to be allotted for community interactions
	Students took keen interest to learn	Provide some time initially for students to develop a rapport with the allotted families
	Patiently listening to what local villagers had to say	To ensure availability of men as well in the households
	Efforts to learn local language and communicate with villagers	Some of the students were not clear about what needs to be done
	Students reassured villagers that they will follow-up for next 5 years	To show students the allotted family on the previous day itself to avoid time delay
	Sharing personal mobile numbers with allotted families to establish a feeling of trust	Not all students are comfortable to interact with community
	Local people opening up and responding to student questions	
Community perception	**Friendly** approach by students	Language barrier
	Talking in local language	Limited time for interaction with students
	**Shared their personal numbers** to get in contact in case of any medical issues	Students were new to me so found it difficult to gel
	Tamil speaking students acted as **translator** for their friends and helped us communicate	I am a shy person and so it is challenging for me to interact with new people
	Gave advice on how to stay healthy by improving personal hygiene	
	Students demonstrated empathy by inquiring about the problems faced by us	
Students’ perception	Clarity of instructions	Language barriers
	Presence of Tamil speaking team members	Cultural differences
	Asking general information and not sensitive details, people were comfortable	Involvement of community members in household chores
	Team work and supporting group members	Reluctance to speak
	Orientation session and training on day 1	Shortage of time
	Practicing before coming to the community	Environment factors (viz. Hot weather)
	Spent initial time on building rapport and then only asked questions	
	Constant support and guidance extended by teachers, postgraduates, and interns	

### Feedback from students on interaction with community

After the completion of interaction of medical students with members of the community, students were asked to give their feedback regarding their experiences on their first interaction with local villagers using a google form. Like community members, even all the medical students responded that they were informed prior about the activities which they were supposed to do in the community. Table 7 demonstrates the wide range of factors that aided and acted as inhibiting factors while students were communicating with the assigned families from the students’ perspective.

### Learnings on interaction with community

**Figure 2:**
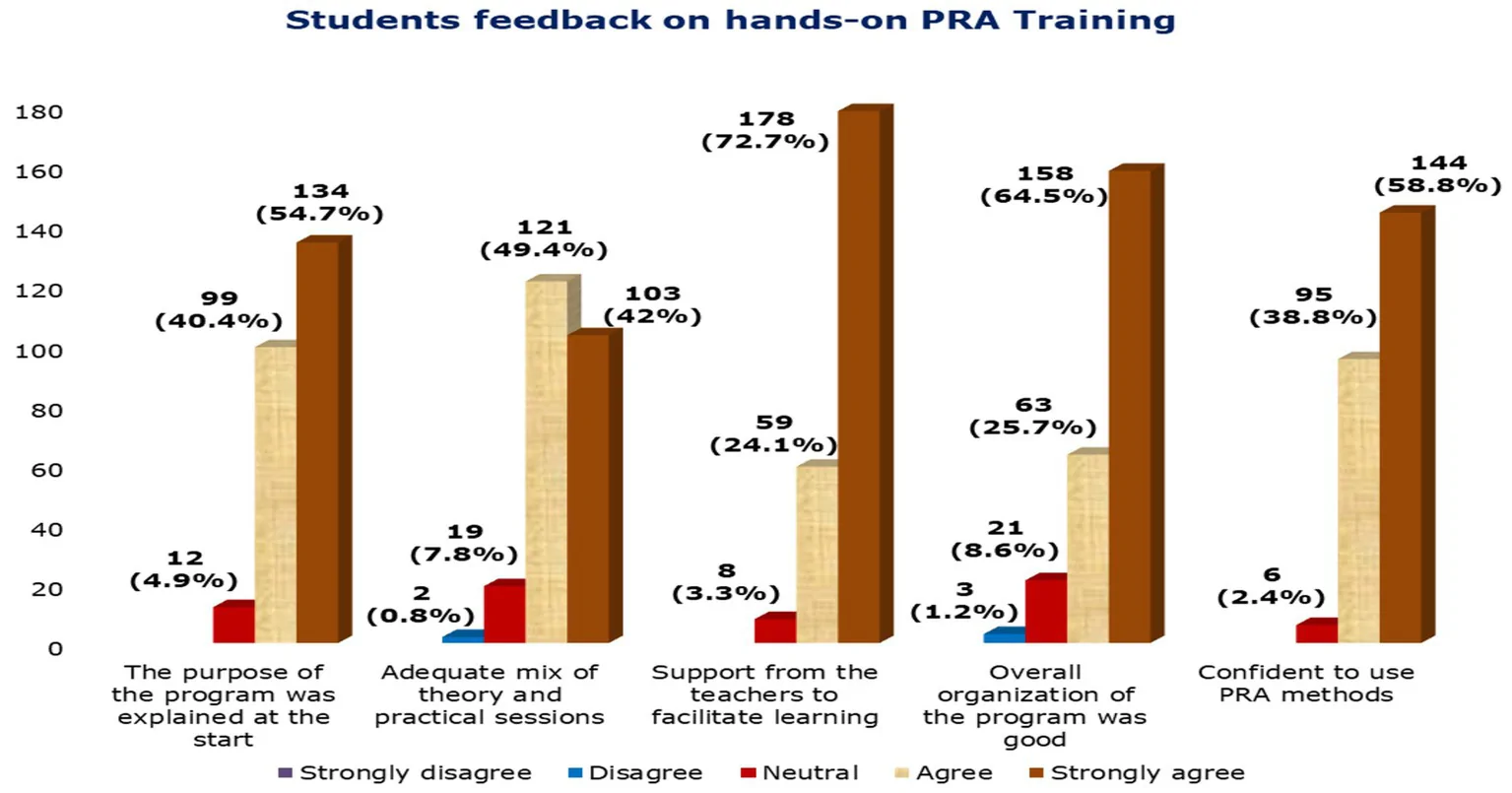
Students’ feedback on hands-on PRA training.

Students reported that they learnt multiple things in Kothimangalam village during their interaction with the residents, such as identification of the sociocultural attributes, lifestyles, common health-related problems, and important landmarks in the village. In addition, students realized the importance of knowing local language, learning the art to establish a rapport, and the importance of group dynamics. Subsequently, a google form was administered to all students to evaluate the entire PRA Training, including the classroom and the community component. Some of these evaluation results have been displayed in Figure 2.

## Discussion

The present study reports that PRA-based training is an effective experiential learning strategy for study participants. The intervention resulted in a substantial improvement in students’ knowledge of PRA methods, while feedback from different stakeholders indicated that PRA encouraged meaningful community engagement, communication, teamwork, and a better understanding of the social determinants of health. These findings indicate that the structured process of integrating CBE into the undergraduate curriculum can help bridge the gap between classroom-based teaching and the practical competencies, thereby strengthening the objectives of CBME. The findings of the study are consistent with Kolb’s experiential learning theory, which advocates that the process of knowledge acquisition becomes more effective when learners actively engage in authentic experiences and apply newly acquired concepts to real-world situations.

In our study, 159(64.9%) students reported that the delivered content significantly increased their knowledge on PRA methods and their understanding of community health. These findings were in alignment with a study done in India that demonstrated that early community orientation program improved students’ communication skills, collaborative learning, and understanding of sociocultural health determinants (23). Similarly, another study reported that early community exposure brought about a significant enhancement in the interest of students towards community medicine, communication skills, teamwork, and attitudes toward community health (24). Together, these findings advocate that structured community-based learning experiences provide authentic opportunities for students to apply theoretical knowledge into real-world public health practices.

In our study, 178 (72.7%) participants appreciated the use of interactive teaching-learning methods in the training program. This preference for interactive teaching methods could be due to the fact that these methods ensure active engagement of the students in the learning process, thereby enabling them to apply theoretical knowledge into practical scenarios (25). In the current study, the responses obtained from study participants suggested that 185 (75.5%) students rated academic proficiency of the teacher on a Likert scale as excellent or very good. The findings obtained from a training workshop done in Iran targeting healthcare workers reported that 30 (62.5%) participants reported the quality of the workshop as excellent (26). The results from another study done in Pakistan reported that 359 (96%) of the trained participants rated workshop as excellent or good (27). These higher rates of academic proficiency of the trainer or quality of the workshop could be attributed to the thorough planning done by the organizing team.

In the present study, 166 (67.8%) students revealed that they liked the training program as the sessions were highly engaging which eventually contributed to joyful learning. In a study done among undergraduate medical students in Egypt, the majority of the workshop participants appreciated the workshop due to the delivery of enjoyable learning experience (28). This preference for the joyful learning could be due to the fact that such an approach significantly enhances the engagement level of students, provides them with the positive learning experience, and improves team building (29). These findings suggest that the effectiveness of the training program extended beyond the quality of instruction and was strongly influenced by the active, community-based learning environment. Further, the collaborative nature of the field activities reduced the teacher-centered hierarchy, thereby facilitating greater student autonomy and appreciation of CBE.

The positive perceptions of students observed in our study are in alignment with findings from various community-based medical education initiatives. In a mixed-methods study, it was reported that a residential community-based training initiative produced remarkable improvements in both cognitive and affective learning domains, suggesting that experiential learning is vital for knowledge acquisition and developing positive attitudes towards community health (30). A qualitative study from Kerala revealed that structured community-based training during the early years of medical training improved students’ confidence, communication skills, and appreciation of community health needs (31). Another cross-sectional study from Bangladesh echoed similar perceptions of medical students regarding community-based teaching experiences (32). All these findings clearly justify the need to actively engage the students in their learning process for better attainment of learning outcomes (25).

It was encouraging to note that 106 (43.3%) of the students liked the training for its professional organization in our study. On a similar note, another training program organized for medical teachers in China, almost all participants rated the organization of event as professional and efficient (33). The improved organization of these training programs is a result of proper planning, systematic approach, positive group dynamics among the team members of the organizing department, keeping the interests of target population in mind, and support from the administration.

In the present study, the assessment of knowledge component demonstrated a statistically significant improvement following the PRA-based training, with the mean average score increasing by 5.33 points on a 10-point assessment scale (from 3.47 to 8.80; *p* < 0.0001). This was accompanied by a very large effect size (Cohen’s *d* = 3.64), suggesting that the intervention produced not only statistically significant but even meaningful gains in knowledge. In a study that was carried out to perform the quantitative and qualitative evaluation of the research methodology workshop, a statistically significant improvement was reported in the areas of manuscript and proposal development (34). The improvement in the mean scores of the study participants could be attributed to the academic competence of the teachers, their ability to engage the students in the session, involvement of interactive teaching-learning methods, and satisfactory clarification of the raised queries.

In our study, we incorporated an additional component of peer observation of teaching, wherein while teachers took their session on PRA methods for first-year undergraduate medical students, the remaining peers (teachers) observed sessions and provided constructive feedback for improvement. Peer observation of teaching has been acknowledged as an effective faculty development and internal quality assurance strategy, with significant impact on reflective teaching practices, improvement in instructional consistency, and sharing of effective teaching approaches (35–38). Moreover, such collaborative approaches can immensely contribute to continuous improvement in educational quality and reinforcement of successful implementation of CBE.

In the current study, teachers reported that students were actively participating in interaction with the community, and they made notes of all the observations in their notebooks. Similar kind of things were encouraged in a study done in Maharashtra, as students were encouraged to make a note of all the observations in the community (16). This act of making note of important observations is like field notes and helps the researcher to recollect the facts once they go back and ensures that none of the essential details are missed. Treating local villagers with respect is a crucial attribute to develop trust and long-term relationship (39, 40). As most of the students were from non-Tamil speaking states, it emerged as a major concern in our study, especially to establish rapport with the residents. When students are unable to communicate directly with community members, interactions often become interviewer-driven, augmenting the risk that community members become passive respondents rather than active partners in identification and analysis of local health needs.

Even though support from interns or bilingual facilitators can help in overcoming immediate communication difficulties, excessive dependence on them significantly reduces opportunities for students to build rapport and engage in quality community interactions. The importance of being aware of the indigenous language has been strongly advocated in a study done among ethnic groups in Mexico (41). Therefore, strategies like pre-placement orientation in the local language, training in cultural competencies, and supervised opportunities for direct community engagement should be given utmost priority, while interpreters or interns should serve only as facilitators, when necessary, rather than acting as substitute for student-community interactions.

In our study, students were asked to do transect walks and interact with the local villagers to know about their problems and invite them for social mapping, but they were not given any checklist to do this, as we wanted them to explore different dimensions related to the lives of rural people. This educational approach was in alignment with the constructivist learning principles, which advocates that learners develop deeper understanding by actively engaging with real learning environments, making independent observations, and construct knowledge via experience and not by adhering to some instructions. On the contrary, in a study done in a village in Maharashtra, students were given a standard checklist to observe the surroundings and explain things to the local people as they were walking across the village (16). However, we cannot ignore that the absence of a structured checklist in our study could have increased cognitive load for these novice students, which could have resulted in some students overlooking important public health infrastructure, environmental, and sociocultural determinants of health. Therefore, while our approach supports learning through autonomy and critical thinking, future programs may benefit from early exploratory visits followed by guided reflection or structured observation tools.

Social mapping in our study was done with the help of local villagers, and they were asked to draw on the ground all important social structures and facilities using locally available sources (13, 14, 42). Force field analysis in our study was done to identify the factors that are promoting and restraining the delivery of healthcare-related services in the identified village. Force field analysis has been constantly employed by different researchers to uncover varied potential themes, like implementation of evidence-based practices in healthcare sector (15); and for promoting adolescents’ involvement in their own health care (11).

One of the important strengths of the present study was the inclusion of feedback from community members, which acknowledged them as active stakeholders in the educational process rather than mere passive recipients of student activities. As we encourage community-engaged medical education, we succeed in developing reciprocal partnerships in which communities contribute to student learning while also benefiting from meaningful interactions with tertiary healthcare institutions. Similar community orientation and rural training programs among undergraduate medical students have highlighted that involvement of community members throughout educational activities helped students in their knowledge acquisition, development of communication skills, and social accountability (23, 24, 30).

As this was the first time for medical students to come out of their usual medical college and hospital settings, from the organizers’ perspective, it is must that we obtain feedback from students. In the present study, the factors that helped students to have an encouraging learning experience include clarity of instructions, teamwork, orientation given on first day, and constant guidance from teachers, postgraduate students, and interns. However, the language barrier, cultural differences, time constraints, and environmental factors, were identified as the major barriers. In-fact, the importance of receiving feedback from students and valuing the same was rated as the most important one in a study done in the United States of America (43). Another study done to evaluate the practice of community and public health nursing justified the need to develop an exclusive standard feedback form for obtaining feedback from students regarding educational experiences (44).

The present study provided key implications for undergraduate medical education within CBME framework. The approach to integrate PRA methods into CBE in the form of PRATHaM program showcased how experiential, learner-centred approaches can facilitate the development of knowledge alongside essential professional attributes. From a theoretical perspective, the findings support experiential and constructivist learning principles by illustrating that authentic community interactions promote meaningful knowledge construction beyond conventional classroom-based instruction. There is a definite need for multicentric studies with longitudinal follow-up component to evaluate the sustainability of these educational outcomes.

This research has its own limitations, including lack of generalizability, as the study was carried out in a single medical institution and its field practice area and potential social desirability bias. Further, due to the limited number of assessors, not all groups were observed in terms of their behavior with the community members. The absence of a control group limits the ability to attribute the observed improvements exclusively to the implemented program. Nevertheless, standardized implementation of the training program, uniform assessment procedures, and triangulation of quantitative and qualitative findings from multiple stakeholders enhanced the robustness of the evaluation. Also, owing to the short duration of the study period, we could not perform a more comprehensive assessment of the program’s effectiveness and long-term outcomes (Kirkpatrick level 4).

## Conclusion

The PRATHaM program demonstrated that integration of PRA methods into undergraduate medical education can aid in establishment of strong conceptual foundation for community-based learning while simultaneously improving students’ knowledge on PRA methods. The combination of structured classroom sessions with experiential field-based activities promoted active engagement, contextual understanding, communication, and collaborative learning, which cumulatively contributed to the development of competencies required for community-oriented medical practice. The initiative aimed to provide equitable opportunities for active participation and authentic community experiences, but field implementation component was impacted by contextual challenges, especially language barriers. Future programs should prioritize language and cultural orientation while preserving the participatory and experiential nature of PRA to optimize educational impact and accomplish meaningful community engagement.

## Data Availability

The raw data supporting the conclusions of this article will be made available by the authors, without undue reservation.

## References

[1] Claramita M Setiawati EP Kristina TN Emilia O van der Vleuten C. Community-based educational design for undergraduate medical education: a grounded theory study. BMC Med Educ. (2019) 19:258. doi: 10.1186/s12909-019-1643-6, 31296217 PMC6624922

[2] Art B De Roo L De Maeseneer J. Towards unity for health utilising community-oriented primary care in education and practice. Educ Health (Abingdon). (2007) 20:74. doi: 10.4103/1357-6283.101614 18058692

[3] Schrewe B Ellaway RH Watling C Bates J. The contextual curriculum: learning in the matrix, learning from the matrix. Acad Med. (2018) 93:1645–51. doi: 10.1097/ACM.0000000000002345, 29979208

[4] Supe A. Graduate medical education regulations 2019: competency-driven contextual curriculum. Natl Med J India. (2019) 32:257–61. doi: 10.4103/0970-258X.295962 32985438

[5] Van der Veken J Valcke M De Maeseneer J Derese A. Impact of the transition from a conventional to an integrated contextual medical curriculum on students’ learning patterns: a longitudinal study. Med Teach. (2009) 31:433–41. doi: 10.1080/01421590802141159, 18825559

[6] Murakami M Kawabata H Kisa K Maezawa M. What rural physicians need to engage in community based education: a qualitative interview survey. J Rural Med. (2012) 7:38–41. doi: 10.2185/jrm.7.38, 25648537 PMC4309331

[7] Sturmberg JP Reid S Khadra MH. A longitudinal, patient-centred, integrated curriculum: facilitating community-based education in a rural clinical school. Educ Health Change Learn Pract. (2002) 15:294–304. doi: 10.1080/1357628021000012787, 14741937

[8] Mahmood MA Khan KS Kadir MM Barney N Ali S Tunio R. Utility of participatory rural appraisal for health needs assessment and planning. JPMA J Pak Med Assoc. (2002) 52:296–300. 12481660

[9] Maalim AD. Participatory rural appraisal techniques in disenfranchised communities: a Kenyan case study. Int Nurs Rev. (2006) 53:178–88. doi: 10.1111/j.1466-7657.2006.00489.x 16879180

[10] Shafaghat T Zarchi MKR Nasab MHI Kavosi Z Bahrami MA Bastani P. Force field analysis of driving and restraining factors affecting the evidence-based decision-making in health systems; comparing two approaches. J Educ Health Promot. (2021) 10:419. doi: 10.4103/jehp.jehp_1142_20 35071625 PMC8719555

[11] MacDuffie H DePoy E. Force field analysis: a model for promoting adolescents’ involvement in their own health care. Health Promot Pract. (2004) 5:306–13. doi: 10.1177/1524839903257994 15228786

[12] Gona JK Xiong T Muhit MA Newton CR Hartley S. Identification of people with disabilities using participatory rural appraisal and key informants: a pragmatic approach with action potential promoting validity and low cost. Disabil Rehabil. (2010) 32:79–85. doi: 10.3109/09638280903023397 19925280 PMC3166842

[13] Chiyaka T Mushati P Hensen B Chabata S Hargreaves JR Floyd S . Reaching young women who sell sex: methods and results of social mapping to describe and identify young women for DREAMS impact evaluation in Zimbabwe. PLoS One. (2018) 13:e0194301. doi: 10.1371/journal.pone.0194301 29543858 PMC5854392

[14] Kathirvel S Jeyashree K Patro BK. Social mapping: a potential teaching tool in public health. Med Teach. (2012) 34:e529–31. doi: 10.3109/0142159X.2012.670321 22452276

[15] McNett M Tucker S Zadvinskis I Tolles D Thomas B Gorsuch P . A qualitative force field analysis of facilitators and barriers to evidence-based practice in healthcare using an implementation framework. Glob Implement Res Appl. (2022) 2:195–208. doi: 10.1007/s43477-022-00051-6 35974880 PMC9373890

[16] Dongre AR Deshmukh PR Garg BS. Using the “transect walk” as a public health teaching and learning tool. Med Educ. (2009) 43:1093–4. doi: 10.1111/j.1365-2923.2009.03483.x, 19874511

[17] Kaye DK Muhwezi WW Kasozi AN Kijjambu S Mbalinda SN Okullo I . Lessons learnt from comprehensive evaluation of community-based education in Uganda: a proposal for an ideal model community-based education for health professional training institutions. BMC Med Educ. (2011) 11:7. doi: 10.1186/1472-6920-11-7, 21362181 PMC3056836

[18] Bamdas JAM Averkiou P Jacomino M. Service-learning programs and projects for medical students engaged with the community. Cureus. (2022) 14:e26279. doi: 10.7759/cureus.26279, 35898383 PMC9308941

[19] Nichols A Worley PS Toms L Johnston-Smith PR. Change of place, change of pace, change of status: rural community training for junior doctors, does it influence choices of training and career? Rural Remote Health. (2004) 4:259. doi: 10.22605/RRH259, 15884989

[20] Shannon CK Baker H Jackson J Roy A Heady H Gunel E. Evaluation of a required statewide interdisciplinary rural health education program: student attitudes, career intents and perceived quality. Rural Remote Health. (2005) 5:405. doi: 10.22605/RRH405 16283824

[21] Walters L Seal A McGirr J Stewart R DeWitt D Playford D. Effect of medical student preference on rural clinical school experience and rural career intentions. Rural Remote Health. (2016) 16:3698. doi: 10.22605/RRH3698 27854470

[22] Dongre AR Deshmukh PR Gupta SS Garg BS. An evaluation of ROME camp: forgotten innovation in medical education. Educ Health. (2010) 23:363. doi: 10.4103/1357-6283.101501, 20589607

[23] Narapureddy BR Patan SK Deepthi CS Chaudhuri S John KR Chittooru C . Development of a community orientation program (COP) as a community-based medical education method for undergraduate medical students: an experience from India. BMC Med Educ. (2021) 21:626. doi: 10.1186/s12909-021-03069-w, 34949199 PMC8697537

[24] Bhattacharrya H Medhi GK Pala S Sarkar A Kharmujai OM Lynrah W. Early community-based teaching of medical undergraduates for achieving better working skills in the community. J Educ Health Promot. (2018) 7:161. doi: 10.4103/jehp.jehp_153_18, 30693298 PMC6332666

[25] Sivarajah RT Curci NE Johnson EM Lam DL Lee JT Richardson ML. A review of innovative teaching methods. Acad Radiol. (2019) 26:101–13. doi: 10.1016/j.acra.2018.03.025, 30929697

[26] Heydari MR Taghva F Amini M Delavari S. Using Kirkpatrick’s model to measure the effect of a new teaching and learning methods workshop for health care staff. BMC Res Notes. (2019) 12:388. doi: 10.1186/s13104-019-4421-y, 31292006 PMC6617554

[27] Savul S Ikram A Khan MA Khan MA. Evaluation of infection prevention and control training workshops using Kirkpatrick’s model. Int J Infect Dis. (2021) 112:76–80. doi: 10.1016/j.ijid.2021.09.005, 34508862

[28] Mostafa SR Khashab SK Fouaad AS Abdel Baky MA Waly AM. Engaging undergraduate medical students in health research: students’ perceptions and attitudes, and evaluation of a training workshop on research methodology. J Egypt Public Health Assoc. (2006) 81:99–118. 17382086

[29] Thesen T Bahner I Belovich AN Bonaminio G Brenneman A Brooks WS . Not just fun and games: game-based learning in health professions education. Med Sci Educ. (2023) 33:1301–6. doi: 10.1007/s40670-023-01859-z, 37886278 PMC10597927

[30] Fathima FN Johnson AR Kiran PR Ratnakumari S Joseph B. Impact of a residential rural community-based training program for medical students on cognitive and affective domains of learning in community medicine: a mixed methods study. Indian J Community Med. (2021) 46:247–51. doi: 10.4103/ijcm.IJCM_545_20 34321735 PMC8281875

[31] Sebastian SR Mathew G Johnson AKS Chacko A Babu BP Joseph MR. Perceptions on the impact of a structured community-based training model in undergraduate medical training during the first phase of clinical exposure: a qualitative study from Kerala. Indian J Public Health. (2021) 65:291–3. doi: 10.4103/ijph.IJPH_1265_20 34558493

[32] Ahmed SMM Hasan MN Kabir R Arafat SMY Rahman S Haque M . Perceptions of medical students regarding community-based teaching experiences: an observation from Bangladesh. Rural Remote Health. (2019) 19:4614. doi: 10.22605/RRH4614, 31400766

[33] Zhao W Liu Z Wang T Yin X Sun Y Zhang X . Assessment of a training project of English as a media of instruction(EMI) using Kirkpatrick model. BMC Med Educ. (2023) 23:271. doi: 10.1186/s12909-023-04204-5, 37081506 PMC10120192

[34] Abdulghani HM Shaik SA Khamis N Al-Drees AA Irshad M Khalil MS . Research methodology workshops evaluation using the Kirkpatrick’s model: translating theory into practice. Med Teach. (2014) 36:S24–9. doi: 10.3109/0142159X.2014.886012, 24617780

[35] Kitto S Danilovich N Rowland P Leslie K Hendry P Hodgson A . Teaching observation as a faculty development tool in medical education: a scoping review. J Contin Educ Heal Prof. (2024) 44:249–59. doi: 10.1097/CEH.0000000000000523 37466351

[36] Stockdill M Hendricks B Barnett MD Bakitas M Harada CN. Peer observation of teaching: a feasible and effective method of physician faculty development. Gerontol Geriatr Educ. (2023) 44:261–73. doi: 10.1080/02701960.2021.2019030, 35196209 PMC9395544

[37] Shaines M Cassese T. Peer observation of teaching program for the busy hospitalist. Cureus. (2022) 14:e26512. doi: 10.7759/cureus.26512, 35923497 PMC9342666

[38] Pedram K Brooks MN Marcelo C Kurbanova N Paletta-Hobbs L Garber AM . Peer observations: enhancing bedside clinical teaching behaviors. Cureus. (2020) 12:e7076. doi: 10.7759/cureus.7076, 32226677 PMC7093940

[39] Khodyakov D Mikesell L Bromley E. Trust and the ethical conduct of community-engaged research. Eur J Pers Cent Healthc. (2017) 5:522–6. doi: 10.5750/ejpch.v5i4.1263, 29375883 PMC5785932

[40] Miranti R Evans M. Trust, sense of community, and civic engagement: lessons from Australia. J Community Psychol. (2019) 47:254–71. doi: 10.1002/jcop.22119, 30125978

[41] Olko J Lubiewska K Maryniak J Haimovich G de la Cruz E Cuahutle Bautista B . The positive relationship between indigenous language use and community-based well-being in four Nahua ethnic groups in Mexico. Cult Divers Ethnic Minor Psychol. (2022) 28:132–43. doi: 10.1037/cdp0000479, 34672647

[42] Marcil L Afsana K Perry HB. First steps in initiating an effective maternal, neonatal, and child health program in urban slums: the BRAC Manoshi project’s experience with community engagement, social mapping, and census taking in Bangladesh. J Urban Health. (2016) 93:6–18. doi: 10.1007/s11524-016-0026-0, 26830423 PMC4794453

[43] Dent MM Boltri J Okosun IS. Do volunteer community-based preceptors value students’ feedback? Acad Med. (2004) 79:1103–7. doi: 10.1097/00001888-200411000-00020 15504781

[44] Reilly JR Collier J Edelstein J Vandenhouten C Hovarter R Hansen JM . Collaborative design and use of an agency feedback form for student clinical practicum experience in community/public health nursing. Public Health Nurs. (2012) 29:160–7. doi: 10.1111/j.1525-1446.2011.00969.x, 22372453

